# Different Roles of Resident and Non-resident Macrophages in Cardiac Fibrosis

**DOI:** 10.3389/fcvm.2022.818188

**Published:** 2022-03-07

**Authors:** Siyuan Hu, Meng Yang, Shumin Huang, Senjie Zhong, Qian Zhang, Haichao Ding, Xiajun Xiong, Zhixi Hu, Yi Yang

**Affiliations:** ^1^School of Sports Art, Hunan University of Chinese Medicine, Changsha, China; ^2^College of Health Science, Wuhan Sports University, Wuhan, China; ^3^Institute of Chinese Medicine Diagnosis, Hunan University of Chinese Medicine, Changsha, China; ^4^Graduate School, Hunan University of Chinese Medicine, Changsha, China

**Keywords:** cardiac fibrosis, resident macrophages, non-resident macrophages, heterogeneity, inflammation

## Abstract

Cardiac fibrosis is a key pathological link of various cardiovascular diseases to heart failure. It is of great significance to deeply understand the development process of cardiac fibrosis and the cellular and molecular mechanisms involved. Macrophages play a special role in promoting heart development, maintaining myocardial cell homeostasis and heart function. They are involved in the whole process from inflammatory to cardiac fibrosis. This article summarizes the relationship between inflammation and fibrosis, discusses the bidirectional regulation of cardiac fibrosis by macrophages and analyses the functional heterogeneity of macrophages from different sources. It is believed that CCR2^–^ cardiac resident macrophages can promote cardiac function, but the recruitment and infiltration of CCR2^+^ cardiac non-resident macrophages aggravate cardiac dysfunction and heart remodeling. After heart injury, damage associated molecular patterns (DAMPs) are released in large quantities, and the inflammatory signal mediated by macrophage chemoattractant protein-1 (MCP-1) promotes the infiltration of CCR2^+^ monocytes and transforms into macrophages in the heart. These CCR2^+^ non-resident macrophages not only replace part of the CCR2^–^ resident macrophage subpopulation in the heart, but also cause cardiac homeostasis and hypofunction, and release a large number of mediators that promote fibroblast activation to cause cardiac fibrosis. This article reveals the cell biology mechanism of resident and non-resident macrophages in regulating cardiac fibrosis. It is believed that inhibiting the infiltration of cardiac non-resident macrophages and promoting the proliferation and activation of cardiac resident macrophages are the key to improving cardiac fibrosis and improving cardiac function.

## Introduction

Cardiac fibrosis is due to excessive activation of cardiac fibroblasts and differentiation into myofibroblasts, which secrete a large number of collagen fibers and excessive deposition, and is accompanied by the death of cardiomyocytes, which results in poor remodeling of the heart. This is the result of the imbalance between collagen catabolism and anabolism, and it is also the pathological response of various end-stage heart diseases. Severe fibrotic reaction will lead to the decline of systolic and diastolic function of the heart, which will destroy the hemodynamic homeostasis, and eventually heart failure will occur (1). Therefore, inhibiting cardiac fibrosis and preventing myocardial remodeling is one of the important ways to prevent and treat cardiovascular diseases.

At the cellular level, cardiomyocytes, fibroblasts, macrophages, endothelial cells and smooth muscle cells can all regulate the occurrence and development of cardiovascular diseases (2, 3). Among them, fibroblasts are the decisive cells in the regulation of cardiac fibrosis. It can differentiate into myofibroblasts, which lead to fibrosis by secreting collagen to deposit ECM. Therefore, fibroblasts are the key to all fibrotic diseases (4), suggesting that any factor affecting cardiac fibroblast transdifferentiation contributes to cardiac fibrosis.

Macrophages, highly heterogeneous cells, are present in all tissues. They have multiple functions and are often used as therapeutic targets for a variety of diseases (5). Macrophages are generally classified into two types, pro-inflammatory (M1-like) and anti-inflammatory (M2-like). Bone marrow-derived inflammatory macrophages are classified as “M1-like” and play an important role in killing pathogens, clearing cellular debris, and in immune responses (6). Embryonic-derived resident macrophages are classified as “M2-like” and play an important role in growth and development, maintenance of tissue function and anti-inflammatory (6). Studies have shown that macrophages are closely related to fibroblasts, and they regulate the entire process of fibrosis production, maintenance and regression by recruiting various chemokines and secreting related mediators (7). When the heart is damaged, bone marrow-derived monocytes recruit and infiltrate into the heart to differentiate into macrophages. They replace the embryonic-derived resident macrophages in the heart, which causes a dramatic change in the macrophage phenotype and promotes undesirable remodeling of the heart (7–10). Reducing the infiltration of bone marrow-derived macrophages into the heart can significantly inhibit the adverse remodeling (9, 11). However, the relationship between different macrophage subsets and cardiac fibrosis is still unclear, and their roles in cardiac fibrosis have not been elucidated (12). Recent studies explain the functional heterogeneity of different macrophage subsets, but present conflicting results on the relationship of macrophages to fibrosis (9, 10). Therefore, interesting changes in the homeostasis of macrophage subpopulations in the heart may be a key target for the development of cardiac fibrosis. This article will discuss the effects and possible mechanisms of different cardiac macrophage subsets on cardiac fibrosis from the progression of inflammatory response and subsequent homeostatic restoration.

## Persistent Inflammation Is the Key to the Formation and Progression of Cardiac Fibrosis

**FIGURE 1:**
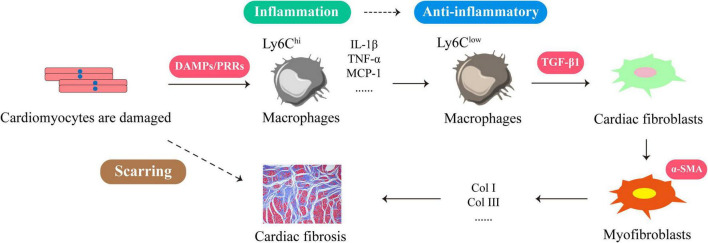
The formation process of cardiac fibrosis is closely related to the inflammatory response. When cardiomyocytes are damaged or necrotic, the cells release large amounts of DAMPs to recruit Ly6C^hi^ macrophages. These DAMPs signal through their membrane PRRs to produce more inflammatory factors and chemokines, such as IL-1β, TNF-α, MCP-1, NF-κB. This will trigger a massive immune response to clear cellular debris and extracellular matrix. A few days later, Ly6C^low^ repairing macrophages emerged at the injury site and produced large amounts of TGF-β1, which stimulated the transdifferentiation of cardiac fibroblasts into myofibroblasts. They secrete Col I and Col III leading to cardiac fibrosis.

The formation and development of cardiac fibrosis is inseparable from inflammation (13, 14). When myocardial cells are damaged or necrotic due to myocardial hypertrophy, myocardial infarction or early heart failure, a large number of damage associated molecular patterns (DAMPs) are released from the cells. They signal through specific receptors such as RAGE, TLR2, and TLR4 for rapid recruitment of neutrophils, cardiac resident macrophages (15, 16). After approximately 30 min, Ly6C^hi^ monocytes infiltrated into the heart via CCR2/CCL2 signaling and differentiated into macrophages. They cause a series of strong aseptic inflammation and produce a series of inflammatory factors and chemokines such as IL-1β, tumor necrosis factor-α (TNF-α), macrophage chemoattractant protein-1 (MCP-1), nuclear factor kappa-B (NF-κB) (17). This will trigger a massive immune response to remove cellular debris and extracellular matrix. After a few days, Ly6C^hi^ monocytes gradually transform into Ly6C^low^ repaired macrophages (18). At this time, a large amount of transforming growth factor-β1 (TGF-β1) is produced, which stimulates the transdifferentiation of cardiac fibroblasts into myofibroblasts. They secrete many fibrous tissues such as type I collagen (Col I) and type III collagen (Col III) to prevent heart rupture. But as the inflammation continues, fibrous tissue overproduces and begins to wrap around and separate the damaged cardiomyocytes. This not only leads to scarring, but also to reduced cardiac compliance and cardiac function (17, 19, 20) (Figure 1). Therefore, timely elimination of inflammation is essential to prevent cardiac fibrosis (21, 22). For example, the use of IL-1 antagonists or knockout of MCP-1 can reduce adverse cardiac remodeling (23, 24). In fact, the production of cardiac fibrosis is also related to the release of anti-inflammatory mediators, among which TGF-β1 is a key anti-inflammatory mediator and an important “switch” for cardiac fibrosis (19, 25). Due to untimely or incomplete resolution of inflammation, the heart continues to be damaged and progressively worsens, which leads to elevated TGF-β1 expression. They stimulate cardiac fibroblast activation, prompting their transformation into myofibroblasts, leading to cardiac fibrosis. Therefore, anti-inflammatory and inhibition of TGF-β1 signaling are effective ways to improve cardiac fibrosis (19, 26).

With the deepening of the research, some contradictory results have emerged. On the one hand, inhibition of the anti-inflammatory factor TGF-β1 at an early stage instead leads to increased mortality, increased neutrophil infiltration, and increased pro-inflammatory cytokines and chemokines (27). This indicates that TGF-β1 is crucial for early cardiac repair, and the effect of inhibiting TGF-β1 signaling in improving fibrosis may be limited to late stages. On the other hand, appropriate inflammation in the early stages of cardiac injury can inhibit cardiac fibrosis. If inflammation is excessively inhibited in this stages, it can adversely affect the heart (28). miR-155 promotes inflammation and can inhibit fibroblast proliferation by increasing inflammation, thereby reducing fibrosis (29). Silencing or inhibiting the expression of miR-155 accelerates the development of fibrosis (30). In addition, some anti-inflammatory drugs can increase the risk of the heart disease. For example, the use of non-steroidal anti-inflammatory drugs (NSAIDs) can promote the development and progression of heart failure (31, 32). Steroids also increase the incidence of cardiac rupture, which is a potential cause of sudden death from cardiac hypertrophy, myocardial ischemia and cardiac remodeling (33).

To sum up, the early inflammation and necessary repair are important for improving cardiac function, while persistent inflammation can cause persistent damage to cardiac tissue, which in turn promotes the development of cardiac fibrosis and the deterioration of cardiac function (34). It suggests that there is a temporal and spatial logical relationship between pro-inflammatory and anti-inflammatory in the process of cardiac injury. Therefore, a certain degree of inflammation is the key to initiating tissue repair, and a proper transition is required between pro-inflammatory and anti-inflammatory. Restraining excessive and persistent inflammation and inhibiting the over-activation of fibroblasts at the appropriate time is one of the effective ways to resolve cardiac fibrosis and achieve optimal repair of tissue and function.

## Macrophages Have Bidirectional Regulation of Cardiac Fibrosis

Although inflammation reveals the formation and progression of cardiac fibrosis, macrophages, as key cells of the immune response, exhibit a unique bidirectional regulatory role in inflammation and fibrosis. In terms of pro-fibrosis, first, bone marrow-derived macrophages are a potential source of fibroblasts and myofibroblasts during fibrosis. In particular, CD206^+^ M2 macrophages can transform to α-SMA^+^ myofibroblasts (macrophage-myofibroblast transition, MMT) through the TGF-β/Smad3 signaling pathway to promote collagen production and aggravate fibrosis (35–37). Second, macrophages can also promote cardiac fibrosis through Ang II and aldosterone (38). Third, bone marrow-derived macrophages can secrete a large number of inflammatory factors (such as IL-1β, TNF-α, IL-6) and pro-fibrotic factors (such as TGF-β, PDGF, FGF) (12). Among them, inflammasomes such as IL-1β, TNF-α, and IL-6 are also essential for the formation of fibrosis (39–41). They induce cardiomyocyte hypertrophy and death, accelerating heart failure (12, 42). Macrophages are also major producers of TGF-β1 (43–45). They induce the transformation of cardiac fibroblasts into myofibroblasts through TGF-β1/Smad signaling pathway and other pathways to promote collagen synthesis and lead to fibrosis (12, 46). For example, CD11b^+^ F4/80^+^ Gr1^+^ monocyte-derived macrophages secrete large amounts of TGF-β1 (47). Ly6C^lo^ non-resident macrophages promote fibrosis, whereas resident macrophages do not (48). Interestingly, whether TGF-β1 is anti-inflammatory or pro-fibrotic can be based on its source. It acts anti-inflammatory if produced by regulatory T cells; but promotes fibrosis if derived from macrophages (7).

**FIGURE 2:**
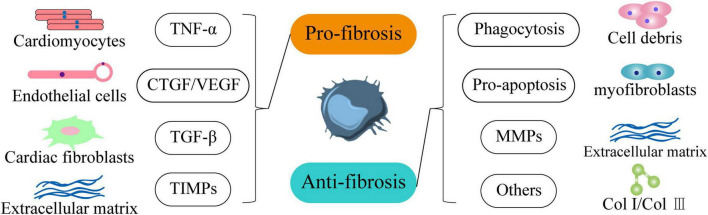
Macrophages regulate the occurrence and development of cardiac fibrosis in both directions. In terms of pro-fibrosis, it promotes the development of heart failure by secreting TNF-α to make cardiomyocytes hypertrophy and accelerate death. Secondly, it stimulates endothelial cells through CTGF and VEGF to indirectly promote fibrosis. Moreover, it activates cardiac fibroblasts through TGF-β1. In addition, it inhibits the degradation of extracellular matrix components through TIMPs. In terms of anti-fibrosis, macrophages can phagocytize cell debris through phagocytic function and promote the apoptosis of myofibroblasts. It also effectively degrades extracellular matrix components through MMPs and inhibits the production of Col I and Col III.

In terms of anti-fibrosis, first, macrophages secrete myofibroblast apoptosis factors to remove apoptotic fibroblasts and other cell debris. Second, macrophages can phagocytose and digest extracellular matrix (ECM) components and reduce the production of inflammatory factors. In addition, Ly-6C^lo^ CD11b^hi^ F4/80^int^ macrophages can not only express a large number of MMPs that degrade collagen (49), but also stimulate other cells to produce MMPs, which is very important for the resolution of fibrosis (7, 49, 50) (Figure 2). In conclusion, macrophages play an important role in cardiac fibrosis through multiple pathways as regulators of fibroblasts. We speculate that the bidirectional regulatory effect of macrophages on cardiac fibrosis is related to different macrophage subpopulations. Therefore, exploring the functional characteristics of different macrophage subsets and elucidating the mechanisms of macrophage phenotypic transformation, differentiation and recruitment can help slow and reverse excessive cardiac fibrosis.

## There are Tissue Resident Macrophages and Monocyte-Derived Non-Resident Macrophages in the Heart

Macrophages are essential in maintaining homeostasis and stress. The traditional view is that tissue macrophages are derived from circulating monocytes, which are divided into classical activated macrophages (M1) and alternative activated macrophages (M2). However, the concept of the M1/M2 macrophage project is derived from *in vitro* studies, which may not reflect the more subtle phenotypes observed *in vivo*. In fact, macrophages cannot form a stable subpopulation, and their response under the influence of various factors will lead to a complex mixed phenotype (5, 38, 51). Therefore, macrophage polarization is more of a normal physiological process, and it may be relatively difficult to study *in vivo*.

Recent studies have found that resident macrophages in the heart already exist before birth (52). The genetic fate map showed that the yolk sac and fetal monocyte progenitor cells produced most of the cardiac resident macrophages. The heart is also one of the few adult organs that retain a large number of yolk sac-derived macrophages. Cardiac resident macrophages derived from the yolk sac persist in adulthood (52), while cardiac non-resident macrophages are formed by infiltration of monocytes from peripheral blood. Therefore, there are two types of macrophages in the heart, one is tissue-resident macrophages, and the other is monocyte-derived macrophages. Resident macrophages are formed by the migration of macrophage precursor cells in the yolk sac during embryonic development, and they have not undergone the monocyte stage. It is also called embryo-derived macrophages, which are innately acquired by the heart. The monocyte-derived macrophages are formed after birth from monocytes in the peripheral blood under the influence of chemokines and inflammatory factors through the CCR2 receptor on the cell membrane to infiltrate and differentiate into the heart during the developmental stage (6, 53, 54). It is also called circulating-derived macrophages or cardiac non-resident macrophages, which are obtained after birth (55). Therefore, the adult heart contains yolk sac-derived CCR2^–^ macrophages and monocyte-derived CCR2^+^ macrophages (that originate from bone marrow progenitors) (56).

**FIGURE 3:**
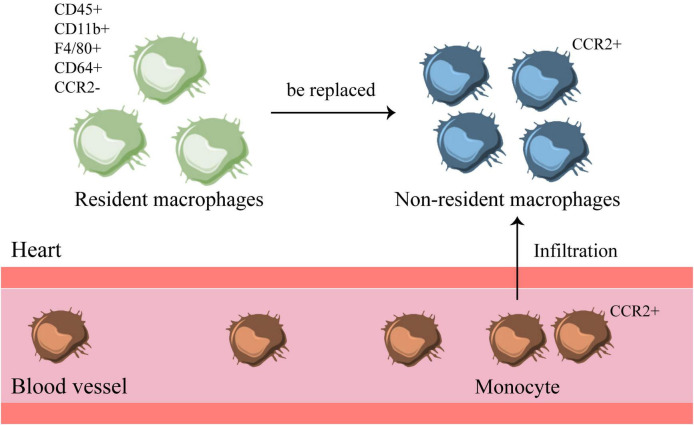
There are resident and non-resident macrophages in the heart. There are two types of macrophages in the heart, one is CCR2^–^ resident macrophages, which exist before birth. The other is non-resident macrophages, which are transformed by CCR2^+^ monocytes in the blood that infiltrate into the heart. When inflammation occurs, monocytes infiltrate the heart and turn into non-resident macrophages, which will replace the original resident macrophages in the heart.

Currently, the heart is considered to be one of the organs that retains large numbers of embryonic macrophages (52). In the mouse heart, macrophages are marked by CD45^+^ CD11b^+^ F4/80^+^ CD64^+^, and their subpopulations are mostly marked by Mac-2, Mac-3, Ly6C, MHC-II, CD11c, CCR2, TIMD4, LYVE1 (10, 57). Among them, CCR2^–^ TIMD4^+^ LYVE1^+^ can be used to recognize yolk sac-derived macrophages (57). Specific subsets of different macrophages are maintained through local proliferation or replacement of circulating monocytes (42, 53, 58) (Figure 3). The research on macrophages from different sources in the heart is still in the initial stage, and it is necessary to explore their functions and effects.

## Resident and Non-Resident Macrophages Have Functional Heterogeneity in Heart Disease

Studies believe that macrophages are essential for tissue regeneration and represent an evolutionary conservative mechanism for tissue repair (59). The development and maturation of the immune system is coupled with the terminal differentiation of cardiomyocytes, and the resident macrophages in the heart are necessary for maintaining heart rhythm and promoting the proliferation of cardiomyocytes (3, 60). Recent studies have shown that the number of macrophages in the atrioventricular node of the heart is not only abundant, but also consistent with the action potential of cardiomyocytes. They are connected by the Cx43 protein, which is indispensable for maintaining the normal rhythm of the heart (60). The latest accelerated review article has received widespread attention and changed people’s perception of cardiomyocyte progenitor cells, because researchers found that it improves heart function through macrophages (61). In addition, cardiac aging is related to the phenotypic changes of resident macrophages, including the upregulation of pro-fibrosis genes, which is one of the main causes of aging-related cardiac fibrosis (62). Generally speaking, a large infiltration of non-resident macrophages will replace the subpopulation of resident macrophages. In fact, even if there is no inflammation, the resident macrophages derived from the yolk sac in the adult heart will gradually be replaced by non-resident macrophages derived from monocytes with age (63). This may indirectly indicate that the risk of heart-related diseases increases with age. In summary, cardiac macrophages play a very critical role in maintaining heart function.

**FIGURE 4:**
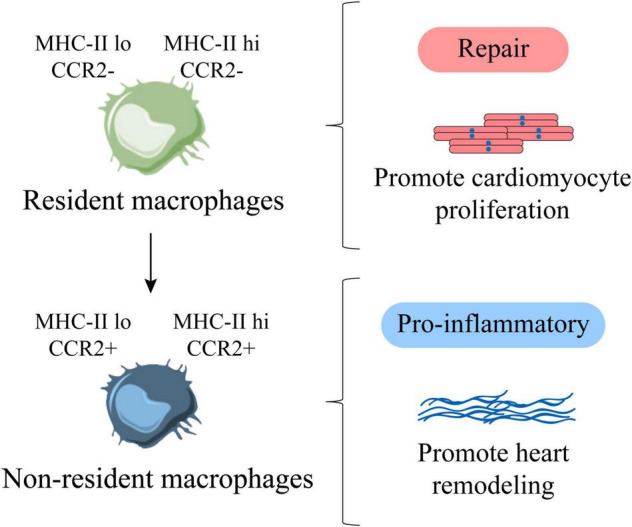
Cardiac resident and non-resident macrophages are functionally heterogeneous. MHC-II^lo^ CCR2^–^ and MHC-II^hi^ CCR2^–^ are cardiac resident macrophages, representing repair macrophages, which are mainly derived from proliferation, which can promote the proliferation of cardiomyocytes. MHC-II^lo^ CCR2^+^, MHC-II^hi^ CCR2^+^ are cardiac non-resident macrophages, representing pro-inflammatory macrophages, which are mainly derived from the infiltration of monocytes, which can promote adverse heart remodeling. Factually, with age, resident macrophages are gradually replaced by non-resident macrophages.

In the study of cardiomyopathy, it was found that most of the pro-inflammatory macrophages were derived from monocytes, while the repair-type macrophages were mainly derived from the resident macrophages of the heart. Moreover, the proliferation ability of cardiac resident macrophages is much higher than that of non-resident macrophages, which indicates that most proliferative macrophages are resident repair macrophages, and most non-proliferative macrophages are derived from pro-inflammatory macrophages infiltrated by monocytes (64). Promoting the proliferation of cardiac resident macrophages has a beneficial effect on improving heart disease, while inhibiting the proliferation of cardiac resident macrophages will aggravate the process of cardiomyopathy (64). In addition, after heart injury, CCR2^+^ monocyte-derived macrophages can produce pro-inflammatory cytokines to promote the development of cardiovascular diseases and strengthen heart remodeling (65). In the absence of CCR2^–^ macrophages, the cardiomyocytes of neonatal mice show limited proliferation ability (8), which indicates that non-resident macrophages accelerate cardiac dysfunction, while resident macrophages can promote cardiomyocyte proliferation and improve heart function (57) (Figure 4).

In recent years, there have been new breakthroughs in the exploration of different macrophage subpopulations in adult and newborn mice. It is believed that there are significant differences in the macrophage subpopulations in their hearts. It has been reported that various subpopulations of macrophages in the adult heart have different recruitment kinetics. MHC-II and CCR2 as the main markers are roughly divided into four subgroups, namely MHC-II^lo^ CCR2^–^, MHC-II^hi^ CCR2^–^, MHC-II^lo^ CCR2^+^, MHC-II^hi^ CCR2^+^ (9, 66). As a matter of fact, the heart of newborn mice is dominated by MHC-II^lo^ CCR2^–^. After adulthood (about 8 weeks of age), cardiac MHC-II^lo^ macrophages produce MHC-II^hi^ macrophages (52, 63). Put it another way, MHC-II^lo^ CCR2^–^ and MHC-II^hi^ CCR2^–^ represent a subgroup of resident macrophages derived from the yolk sac from newborn to adulthood, which is born with the heart. Additionally, macrophages are the key to support neonatal heart regeneration (3), which shows that MHC-II^lo^ CCR2^–^ and MHC-II^hi^ CCR2^–^ resident macrophage subpopulations contribute to the proliferation of cardiomyocytes (Figure 4).

When the heart is damaged, the number of macrophages in both the adult heart and the neonatal heart increases significantly, but the increased macrophage subpopulations are not the same. The increased macrophages in adult mice are mostly MHC-II^hi^ CCR2^+^ macrophages derived from circulating monocytes, while the increased macrophages in neonatal mice are caused by the proliferation of MHC-II^lo^ CCR2^–^ macrophages derived from the yolk sac. There is no additional recruitment of circulating pro-inflammatory CCR2^+^ monocytes (9). This reflects the unique role of neonatal heart in maintaining heart function and anti-inflammatory, which shows that resident macrophages are related to cardiomyocyte regeneration and tissue repair (55).

## Resident and Non-Resident Macrophages Regulate the Occurrence and Development of Cardiac Fibrosis

**FIGURE 5:**
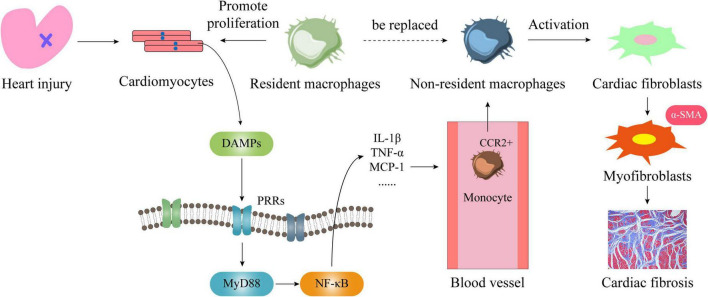
The mechanism of cardiac resident and non-resident macrophages in regulating cardiac fibrosis. When the heart is damaged or cardiomyocytes die, large amounts of DAMPs are released from the nucleus. They combine with PRRs on the cell membrane to activate the MyD88/NF-κB signaling pathway and produce a large number of inflammatory factors and chemokines. Among them, MCP-1 will bind to the monocyte membrane receptor CCR2, so that it will be infiltrated into the heart and become non-resident macrophages. At this time, on the one hand, cardiac resident macrophages are replaced by cardiac non-resident macrophages, on the other hand, cardiac fibroblasts are activated by it to transform into myofibroblasts, and then a large number of collagen fibers are produced, which leads to the heart fibrosis.

Since cardiac resident and non-resident macrophages have different sources, they also have different roles in the occurrence and development of cardiac fibrosis. First of all, endogenous danger signals will appear when cardiomyocytes are damaged or necrosis caused by various heart diseases. DAMPs, such as HMGB1, are released in large quantities, so that pattern recognition receptors (PRRs) are activated and expressed. After PRRs and DAMPs are combined on the cell membrane of inflammatory cells, it produces a large number of inflammatory cytokines through MyD88 or NF-κB signaling pathways to trigger inflammation, including TNF-α, MCP-1, IL-1β, and so on. Secondly, circulating monocytes express CCR2 and are recruited around blood vessels (66). Through the interaction of MCP-1 and the surface receptor CCR2, monocytes infiltrate the heart to derive non-resident macrophages, and they will replace most of the resident macrophages in the heart. Within 24 h of heart injury, neutrophils will rapidly recruit in the injured area. Then Ly-6C^hi^ CCR2^+^ CX3CR1^lo^ monocytes are recruited and infiltrated and differentiated into macrophages to enter the injured area, produce an inflammatory response, remove dead cardiomyocytes and cell debris through proteolysis and phagocytosis (67). Neutrophils disappear in 3–7 days, and repairing macrophages follow. They reduce inflammation, promote angiogenesis and cardiac repair (68). Macrophage colony stimulating factor and a variety of chemokines stimulate these macrophages to secrete mediators that promote fibroblast activation, such as TGF-β1, PDGF, IL-10, VEGF, and so on. These can transform fibroblasts into myofibroblasts, and produce many Col I, Col III, α-SMA, etc., which lead to cardiac scar formation and myocardial remodeling, resulting in cardiac dysfunction (34, 68–72). Moreover, these CCR2^+^ macrophages are rich in NLRP3 inflammasomes, which can secrete large amounts of IL-1β through NLRP3 (52). This further exacerbates the level of inflammation in the heart (73). Therefore, in some cases CCR2 antagonists are used to treat multiple diseases (67). It has also been observed from the histological level that a lot of macrophages accumulate in the heart when injury occurs (74), and the level of inflammatory factors is closely related to the severity of heart failure (75, 76). In addition, the excessive activation of the renin-angiotensin-aldosterone system can enhance the ability of monocytes to migrate and infiltrate the heart by upregulating the expression of IL-6 (77), which causes an increase in the number of non-resident macrophages in the heart. It induces the increase of myofibroblasts and stimulates the synthesis of extracellular matrix to promote scar formation. Therefore, left ventricular systolic dysfunction, poor heart remodeling, and even heart failure is closely related to the massive infiltration of non-resident macrophages (Figure 5).

Unlike CCR2^+^ cardiac non-resident macrophages, mouse cardiac resident macrophages can directly migrate to the injured site and inhibit fibrosis after ischemic injury (78). But if resident macrophages are cleared, adverse cardiac remodeling is promoted (57). It has been reported that CCR2^–^ cardiac resident macrophages express more growth factors such as IGF1, PDGF-C, EGFL7, GDF15, NRP1, SLIT3, ECM1, SDC3, SCN9A, FGF13, etc (79–81). This suggests that CCR2^–^ cardiac resident macrophages maintain cardiac function and inhibit the hyperactivation of cardiac fibroblasts by expressing more growth mediators, which represent a repair phenotype. While CCR2^+^ cardiac non-resident macrophages express more inflammatory factors, chemokines, chemokine receptors, pro-fibrotic factors and hypertrophic factors, such as IL-1, NF-κB, IL-6, IL-1β, CCL7, AREG, EREG, OSM, PTX3, etc (8). This suggests that CCR2^+^ cardiac non-resident macrophages express more inflammatory mediators, which represent an inflammatory phenotype and promote excessive fibrosis. Non-resident macrophages infiltrate massively and continuously replace resident macrophages during cardiac injury caused by myocardial infarction, long-term hypertension or other diseases. This led to a dramatic decrease in the number of resident macrophages in the heart (82), and a marked increase in the number of non-resident macrophages, which resulted in disruption of myocardial homeostasis and inhibition of cardiac function (10, 52). Therefore, inhibiting the infiltration of CCR2^+^ cardiac non-resident macrophages and promoting the proliferation and activation of CCR2^–^ cardiac resident macrophages is an important way to prevent excessive cardiac fibrosis, resolve adverse cardiac remodeling and improve heart failure.

## Concluding Remarks and Prospectives

This article summarizes the occurrence and development of cardiac fibrosis and the source and function of two different macrophages in the heart, as well as their different effects on cardiac fibrosis. It shows that the resident macrophages in the heart have beneficial effects on improving cardiac fibrosis and enhancing cardiac function, and preventing the infiltration of monocyte-derived non-resident macrophages can reduce excessive inflammatory response and facilitate heart repair. Therefore, we believe that the difference in the effects of resident and non-resident macrophages on cardiac fibrosis-related signaling pathways and the interrelationship with fibroblast transdifferentiation determine their roles in cardiac fibrosis. Furthermore, it is unclear whether the recognition and response of DAMPs by related factors in two different sources of macrophages is the key to the functional heterogeneity of the two, and the functional difference between MHC-II^hi^ and MHC-II^lo^ in CCR2^–^ macrophages is still unknown, and the functional heterogeneity of the two and the specific markers of their subgroups are worthy of in-depth study and discussion.

## Author Contributions

SiH conceived the study and wrote most parts of the manuscript. YY and ZH provided methodological support in the study design and revised the manuscript. MY, ShH, SZ, QZ, and XX searched and analyzed some literature. HD participated in the production of the picture. All authors have read and approved the manuscript before submission.

## Conflict of Interest

The authors declare that the research was conducted in the absence of any commercial or financial relationships that could be construed as a potential conflict of interest.

## Publisher’s Note

All claims expressed in this article are solely those of the authors and do not necessarily represent those of their affiliated organizations, or those of the publisher, the editors and the reviewers. Any product that may be evaluated in this article, or claim that may be made by its manufacturer, is not guaranteed or endorsed by the publisher.
